# The influence of stomatal morphology and distribution on photosynthetic gas exchange

**DOI:** 10.1111/tpj.14560

**Published:** 2019-11-10

**Authors:** Emily L. Harrison, Lucia Arce Cubas, Julie E. Gray, Christopher Hepworth

**Affiliations:** ^1^ Department of Molecular Biology and Biotechnology University of Sheffield, Western Bank Sheffield UK

**Keywords:** stomata, photosynthesis, diffusion, gaseous exchange, carbon dioxide, morphology, development, distribution, *Arabidopsis thaliana*

## Abstract

The intricate and interconnecting reactions of C_3_ photosynthesis are often limited by one of two fundamental processes: the conversion of solar energy into chemical energy, or the diffusion of CO_2_ from the atmosphere through the stomata, and ultimately into the chloroplast. In this review, we explore how the contributions of stomatal morphology and distribution can affect photosynthesis, through changes in gaseous exchange. The factors driving this relationship are considered, and recent results from studies investigating the effects of stomatal shape, size, density and patterning on photosynthesis are discussed. We suggest that the interplay between stomatal gaseous exchange and photosynthesis is complex, and that a disconnect often exists between the rates of CO_2_ diffusion and photosynthetic carbon fixation. The mechanisms that allow for substantial reductions in maximum stomatal conductance without affecting photosynthesis are highly dependent on environmental factors, such as light intensity, and could be exploited to improve crop performance.

## Significance Statement

Plant photosynthesis relies on diffusion of CO_2_ from the atmosphere to the chloroplasts through the stomatal pores. The distribution and morphological characteristics of stomata influence this and are of particular importance when attempting to understand or improve on rates of carbon fixation. Nonetheless, alterations expected to affect stomatal conductance do not always cause corresponding alterations in photosynthetic carbon assimilation. This review explores the factors that are likely to cause this observed disconnect between maximum stomatal conductance and photosynthesis.

## Introduction

**Figure 1:**
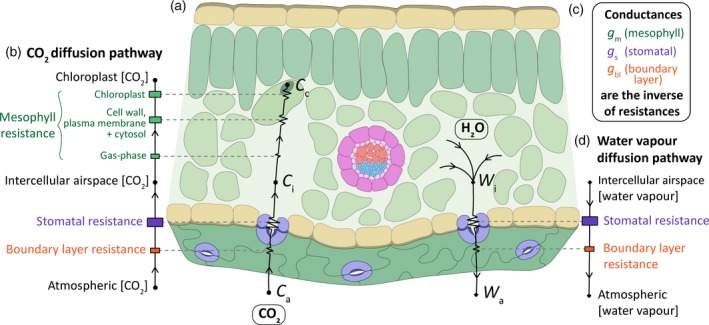
The pathway of diffusive resistance for CO_2_ entry and H_2_O exit in a C_3_ leaf. (a) Diagram of a leaf cross‐section illustrating the route of gaseous exchange. CO_2_ diffuses into the leaf along a concentration gradient from the atmosphere (*C*_a_) into the intercellular airspaces of the leaf (*C*_i_), before finally reaching the chloroplast (*C*_c_). In contrast, water vapour diffuses out of the leaf along a concentration gradient from the intercellular airspaces (*W*_i_) to the atmosphere (*W*_a_). (b) The CO_2_ diffusion pathway comprises a series of resistances, indicated between (b) and (a) via dashed lines. To summarise, CO_2_ diffuses from the atmosphere through a boundary layer of air surrounding the leaf and enters the substomatal cavity via the stomatal pore, encountering boundary layer and stomatal resistance, respectively. CO_2_ must then diffuse through the intercellular airspaces and into the mesophyll cell, encountering gas‐phase resistance, followed by mesophyll cell wall, plasma membrane and cytosol resistances. Finally, CO_2_ diffuses into the chloroplast, encountering chloroplast envelope and stroma resistances, before reaching RuBisCO, where it is used as a substrate in the Calvin–Benson cycle. The size of the boxes represent the assumed magnitude of resistance. (c) The inverse of these resistances are termed conductances, with the corresponding pair shown in the same colour. Boundary layer conductance (*g*_bl_) is shown in orange, stomatal conductance (*g*_s_) is shown in purple and mesophyll conductance (*g*_m_) is shown in green (which corresponds to the collective resistances that exist between gas‐phase and chloroplast). (d) Similarly, the water vapour diffusion pathway encounters a series of resistances. Water vapour diffuses from the intercellular airspaces and out into the atmosphere, encountering stomatal and boundary layer resistances along the way. Again, the corresponding resistances are linked between (c) and (a) via dashed lines.

Life on Earth depends on photosynthesis, the source of our food, oxygen and the overwhelming majority of our energy. Photosynthesis is comprised of two distinct but intimately coupled sets of reactions: the light reactions that produce NADPH and ATP, and the carbon fixation reactions (Calvin–Benson cycle), which utilises them. For photosynthetic carbon fixation (*A*) to occur, CO_2_ must first diffuse from the atmosphere into the interior of the leaf. This fundamental process is made possible in vascular land plants by the presence of stomata: microscopic pores in the epidermal leaf surfaces. As illustrated in Figure 1(a), these pores facilitate the passage of gaseous CO_2_ through the cuticle of the epidermis, into the intercellular airspaces of the leaf, before diffusing into the chloroplast in which CO_2_ is fixed by the carboxylating enzyme RuBisCO. Despite the role of stomata, a significant difference exists between the concentration of atmospheric CO_2_ (*C*_a_) and the relatively low concentrations of CO_2_ within the intercellular airspaces (*C*_i_) and the chloroplast (*C*_c_). This CO_2_ gradient arises through the photosynthetic consumption of CO_2_ in the chloroplast, but also via several sources of resistance within the CO_2_ diffusion pathway (Evans and von Caemmerer, 1996; Evans *et al.*, 2009; Figure 1b). Gaseous CO_2_ must diffuse from the atmosphere across a boundary layer of air that hugs the leaf surface (boundary layer resistance) and into the substomatal cavities via the stomatal pore (stomatal resistance). Once inside the leaf, it must pass through the intercellular airspaces before reaching the mesophyll cell wall, in which CO_2_ dissolves in the water‐filled pores, entering the liquid phase. From here, CO_2_ diffuses across the plasma membrane, enters the cytosol, and diffuses through the chloroplast envelope and stroma, before finally reaching RuBisCO (collectively, this last stage is known as mesophyll resistance). The inverse of these sources of resistance are termed boundary layer conductance (*g*_bl_), stomatal conductance (*g*_s_), and mesophyll conductance (*g*_m_), with the latter two usually providing the greatest contributions to CO_2_ conductance (Figure 1c). Stomata facilitate not only the uptake of CO_2_ but also the exchange of all gases between the aerial parts of the plant and the atmosphere. Water vapour and oxygen molecules exit the leaf through the same path as CO_2_ enters, but usually in the opposite direction (Figure 1a). In a process known as transpiration (*E*), water diffuses from the wet cell surfaces surrounding the intercellular airspaces through the stomatal pore and out into the drier bulk air surrounding the leaf; this flow is again influenced by boundary layer and stomatal resistance (Figure 1d).

A significant limitation to plants with C_3_ photosynthesis, which lack a carbon concentrating mechanism, is the supply of CO_2_ to the site of carboxylation (Farquhar *et al.*, 1980). Another way of conceptualising this is that C_3_ photosynthesis is often limited by its ability to draw down CO_2_ from the atmosphere to the chloroplast resulting in subsaturating *C*_c_, and therefore less CO_2_ substrate available for *A.* Maximising *g*_s_ is an effective way in which plants can increase both *C*_i_ and, via an increase in internal concentration gradients, *C*_c_. This, in turn, increases *A* (Caemmerer and Evans, 1991; Lloyd *et al.*, 1992). However, due to the shared diffusion pathway, increasing *g*_s_ increases not only *C*_i_ but also *E.* This somewhat paradoxical relationship results in plants needing to balance *A* with *E*, with this ratio being referred to as the intrinsic water use efficiency of the plant (*iWUE*). This balance is achieved primarily through two stomatal‐driven mechanisms; either the short‐term adjustments to stomatal pore size (stomatal aperture) or long‐term developmental regulation of stomatal density *(D)*, size (*S*) and patterning.

This review focuses on the role that stomata play in photosynthetic gaseous exchange and is divided into sections describing the effects of stomatal morphology and stomatal distribution on *A*. However, it is important to note that stomatal characteristics are often interlinked and functionally coordinated. For example, *S* frequently dictates *D,* whilst changes in *D* often drive stomatal clustering*.* Thus, at its simplest, stomata affect *A* via changes to *g*_s_; however, the mechanisms that drive these changes are often complex and interconnected. We also explore how recent advances in our understanding of stomatal development and functioning are facilitating attempts to enhance photosynthesis, whilst examining the current limitations of this work.

## The influence of stomatal morphology on gaseous exchange

Each individual stomate is formed of two specialized cells known as guard cells (GCs), which surround the stomatal pore. By dynamically controlling their pore apertures, plants can regulate their gaseous exchange as the first response to a plethora of environmental changes, including light intensity, *C*_a_, temperature, humidity and water availability (Hetherington and Woodward, 2003; Shimazaki *et al.*, 2007). These stomatal movements are mediated by changes in volume and turgor pressure of the GCs, driven by ion exchange and water fluxes across the GC plasma membrane and tonoplast (Kollist *et al.*, 2014). Stomata come in many shapes and sizes, and this can directly affect their functionality through altered transport and accumulation of ions and osmotic solutes or restricted lateral movements of the cell wall (Woolfenden *et al.*, 2018). This, in turn, can affect stomatal dynamism and the total range of movement, impacting on the availability of carbon for photosynthesis*.*

### Stomatal shape

Despite similar turgor‐driven movements, the stomata of vascular land plants are morphologically diverse. Of particular note are the stark differences between eudicots and monocot grass species. The stomatal complexes of many eudicots, such as *Arabidopsis thaliana*, are often regarded as the archetypal form with kidney‐shaped GCs that lack subsidiary cells (anomocytic). Contrastingly, grasses form dumbbell‐shaped GCs, which are flanked by two paracytic subsidiary cells. The mechanical characteristics and subsequent functionality of these diverse stomatal forms have been previously explored. For instance, it has been suggested that the slim linear shape of dumbbell GCs, and thus their lower GC volumes, requires a comparatively smaller change in turgor pressure to generate the same or greater increase in stomatal aperture. They are therefore considered to be more efficient, by requiring the exchange of fewer molecules of water, ions and osmolytes (Raschke, 1976; Hetherington and Woodward, 2003). Franks and Farquhar (2007) demonstrated this by comparing two monocot species whose stomatal complexes both displayed subsidiary cells, but had differently shaped GCs. They confirmed that the dumbbell‐shaped stomata of grass species wheat (*Triticum aestivum*) could indeed open comparatively wider and achieve greater *g*_s_ than the kidney‐like shaped stomata of a non‐grass species. Similarly, when comparing between anomocytic kidney‐shaped stomata, GC shape and thus corresponding GC volume significantly impacted gaseous exchange, with the ‘thinner’ GCs of a fern species facilitating three times higher photosynthetic rates, compared with the ‘fatter’ stomata from an ancient vascular plant taxon (Franks, 2006).

In addition to increased gas exchange capacity, several studies have reported that dumbbell‐shaped stomata display rapid stomatal movements, in which the rate of stomatal opening is at least an order of magnitude faster than in kidney‐shaped stomata (Franks and Farquhar, 2007; Drake *et al.*, 2013; McAusland *et al.*, 2016; Chen *et al.*, 2017; Raissig *et al.*, 2017). These so‐called ‘speedy’ stomata are believed to enable greater optimisation of gas exchange under fluctuating environmental conditions (McAusland *et al.*, 2016; Lawson and Vialet‐Chabrand, 2019). For example, plants with highly responsive stomata may be better able to utilise transient periods of high light (e.g. sunflecks), by opening quicker and thus increasing CO_2_ uptake when photosynthesis is carbon limited. Alternatively, the same plants may reduce unnecessary water loss during sudden periods of low light (e.g. cloud cover) through faster stomatal closure when photosynthesis becomes light limited. Fluctuating light might better represent the dynamism of light availability under field conditions. However, most studies exploring the relationship between stomatal development or behaviour and photosynthesis have been conducted under non‐fluctuating (square wave) light. This represents a gap in our current knowledge that recent papers have begun to address (Vialet‐Chabrand *et al*., 2017a,b; Matthews *et al.*, 2018).

The presence of flanking subsidiary cells are thought to be a significant factor in explaining the enhanced responsiveness of dumbbell‐shaped stomata in grasses (see Nunes *et al.*, 2019, in this issue). These cells facilitate extensive lateral movement of the GCs, regardless of GC shape, through substantial spatial displacement and physical interaction, thus facilitating much greater pore apertures that in turn increase *A* (Franks and Farquhar, 2007). It is suggested that subsidiary cells and GCs are also able to rapidly transfer ions and turgor pressure between each other during stomatal opening and closure, resulting in quicker response times (Raschke and Fellows, 1971). Recent transgenic work in the grass *Brachypodium distachyon* has experimentally confirmed the importance of subsidiary cells in stomatal function. *Brachypodium *plants with a mutation in the stomatal development gene *BdMUTE* failed to recruit subsidiary cells during stomatal formation. These plants were impaired in both the magnitude and speed of response of *g*_s_ to changing light intensity, and although not reported, presumably *A* as well (Raissig *et al.*, 2017). It is believed that the dynamic nature of gramineous stomata could have assisted in the spread and diversification of the grasses, especially in arid environments (Hetherington and Woodward, 2003). In the case of kidney‐shaped GCs, it may be that factors other than those driven by GC morphology are more critical to the rate of the stomatal response, for example, biochemical or mechanical limitations (Lawson and Blatt, 2014; McAusland *et al.*, 2016; Carter *et al.*, 2017). There is room yet for further studies into the broad diversity of stomatal forms and their functions, such as the interaction between subsidiary cells and non‐dumbbell‐shaped GCs, which vary in their number and positioning of subsidiary cells.

### Stomatal size

Whilst the overall shape of stomata is pre‐determined based on species, many species are able to adjust both their *S* and *D*, in order to modify their gas exchange to suit the prevailing conditions. The maximum potential stomatal conductance of a leaf (*g*_smax_), assuming all stomata are fully open, is calculated using empirical measurements of *D*, maximum stomatal pore area (*a*_max_) and stomatal pore depth (*l*). *S* defines both *a*_max_ and *l*, through estimations of GC width and GC length, respectively. Ultimately, *S* and *D* dictate the theoretical capacity for gas exchange with, in principle, infinite combinations of either of the two parameters achieving the same *g*_smax_ (Franks *et al.*, 2015). Although anatomically possible, plants do not operate close to their *g*_smax_, unless subjected to extreme environmental permutations; their operating *g*_s_ instead usually remains at *c.* 20% of their maximum capacity, which corresponds to the turgor pressure in which GCs can most efficiently control pore apertures (Franks *et al.*, 2012; Dow *et al.*, 2014a).

When environmental conditions shift a plant's operating *g*_s_ away from its optimal range over a consistent period, leaf primordia may respond by changing *g*_smax_ via developmental alterations to both *S* and *D*. A negative relationship has often been observed between *S* and *D*, whereby increased *D* is accompanied by a reduction in *S*. This allows plants to pack in larger numbers of stomata without altering the fraction of the epidermis that is allocated to stomata: a spatial constraint that exists to prevent the issues associated with stomata in close proximity or direct contact with each other (de Boer *et al.*, 2016), as discussed later. This phenomenon has been documented across a wide range of species, and geological and evolutionary timescales, suggesting the relationship between these two parameters occurs both as a short‐term plastic response, and a long‐term evolutionary adaptation to the changing environment (Dilcher, 2000; Ohsumi *et al.*, 2007; Franks and Beerling, 2009; Doheny‐Adams *et al.*, 2012). For example, during a period of falling *C*_a_ in the Palaeozoic era, plants exhibited greater numbers of smaller stomata, likely caused by the selection pressure to increase *g*_smax_ and thus maintain *A* under lower CO_2_ (Crowley and Berner, 2001). Whilst reducing *S* alone would, in fact, reduce *g*_smax_, the concomitant increase in *D* leads to an overall increase in photosynthetic potential. The decrease in *S* also causes a reduction in pore depth, due to a smaller cross‐sectional area of the GCs, thus creating a shorter distance for CO_2_ diffusion into the interior of the leaf (Franks and Farquhar, 2007; Franks and Beerling, 2009). Under elevated CO_2_ concentrations, *S* generally increases whilst *D* decreases. These observations imply that under conditions in which a lower *g*_s_ is sufficient for optimum *A*, such as at higher *C*_a_, fewer but larger stomata are more beneficial to the plant. This suggests a cost to the production of high *D* small stomata, which if not compensated for by increased *A*, may be deleterious to plant performance. Firstly, energy is required for the operation and maintenance of each stomate (Assmann and Zeiger, 1987), and higher rates of GC respiration would be expected in plants with high levels of *g*_s_ (Srivastava *et al.*, 1995)*.* Additionally, a reduction in *S* may cause changes in cell wall stiffness and GC mechanics (Carter *et al.*, 2017). Further work is necessary to quantify the demand that alterations to *D* place on the leaf and the specific advantages that large *S* and low *D* may afford to plants.

With the above in mind, *S* arguably influences *A* more through alterations to stomatal responsiveness, than through adjustments in g_smax_. The presence of smaller stomata is generally reported to accelerate stomatal aperture responses compared with larger stomata, due to a greater membrane surface area to GC volume ratio, which increases the rate of ionic fluxes (Hetherington and Woodward, 2003; Franks and Beerling, 2009; Drake *et al*., 2013). It has been shown across a range of species that slow stomatal kinetics reduce *A* by an average of 10% (McAusland *et al.*, 2016); this reduction in carbon gain throughout the day would likely negatively effect yield (Taylor and Long, 2017). Thus, smaller stomata can promote greater photosynthetic rates, particularly under fluctuating environmental conditions (Schlüter *et al.*, 2003; Drake *et al.*, 2013; Tanaka *et al.*, 2013; Lawson and Blatt, 2014). However, the relationship between *S* and stomatal speed is not conserved over wide‐ranging species (Elliott‐Kingston *et al.*, 2016; Haworth *et al.*, 2018) and may be dependent on the physical shape and constraints of the GC, as discussed above. It is important to note that, as *S* and *D* are so intrinsically linked, it is often difficult to distinguish whether improvements in *A* are due to increases in *g*_smax_ via higher *D,* or due to rapid stomatal responses promoted by smaller *S*. The size of stomata is also often positively correlated with overall cell size. For example, increasing genome size or ploidy has been linked to an increase in *S* and a reduction in *D* (Mishra, 1997; Lomax *et al.*, 2009), owing to the larger GC nucleus size (Franks *et al.*, 2012). This may also alter the size of the epidermal pavement cells, which may change the ion reservoirs and mechanical environment supplied to the GCs that could affect stomatal movements. The underlying genetic mechanisms that control *S* are, however, currently unclear. Efforts to manipulate *D* have also affected *S*, thus, no genetically manipulated plants which target *S* alone are available (Doheny‐Adams *et al.*, 2012). This may be due to the inability to uncouple overall cell size and GC size, as facilitating a change in *D,* might also require a change to the size of the pavement cells. Further research is required to uncouple these two parameters and improve our understanding of how *S* directly impacts on *A.*

## Stomatal distribution and its impact on photosynthesis

As we have explored, stomata play a critical role in the exchange of gases and thus to whole plant physiology. It is, therefore, unsurprising that plants have evolved a sophisticated developmental programme to ensure their correct formation and distribution. Early in leaf development, a subset of dispersed protodermal cells divide asymmetrically to each produce a small stem‐cell like meristemoid. These meristemoids either undergo a further asymmetric division or progress through a guard mother cell intermediate, before dividing symmetrically and differentiating into the pair of GCs that surround each stomatal pore. Several core basic helix–loop–helix class transcriptional regulators [SPEECHLESS (SPCH), MUTE and FAMA in conjunction with SCREAM1 and SCREAM2] first identified in the model plant Arabidopsis, act sequentially to control key cell division and differentiation steps involved in the formation of stomata. A central feature of stomatal development, common to many plant species, is the ‘one‐cell spacing rule’. This dictates that stomata are separated from each other by at least one intervening pavement cell in the leaf epidermis and, individually, overlay a single substomatal cavity within the mesophyll layer (Geisler *et al.*, 2000; Peterson *et al.*, 2010; Pillitteri and Dong, 2013). A suite of gene products are involved in maintaining the correct spacing of stomata by modulating the transcriptional activity of SPCH, and thus entry of cells into the stomatal lineage. These include extracellular peptide ligands (EPIDERMAL PATTERNING FACTOR (EPF) family) and their cell‐surface receptor components (ERECTA (ER) family, TOO MANY MOUTHS (TMM) and SOMATIC EMBRYOGENESIS RECEPTOR KINASES) along with a mitogen‐activated protein kinase (MAPK) cascade [see review by Zoulias *et al.* (2018) for a comprehensive review of stomatal development]. Together, this complex signalling network conserved across evolutionary timescales (Liu *et al.*, 2009; Chater *et al*., 2016; Hepworth *et al.*, 2018), provides multiple levels of regulation that permits a high degree of plasticity and allows plants to adjust their final number and pattern of stomata, in response to environmental conditions and internal cues (Casson and Gray, 2008).

### Stomatal density

**Figure 2:**
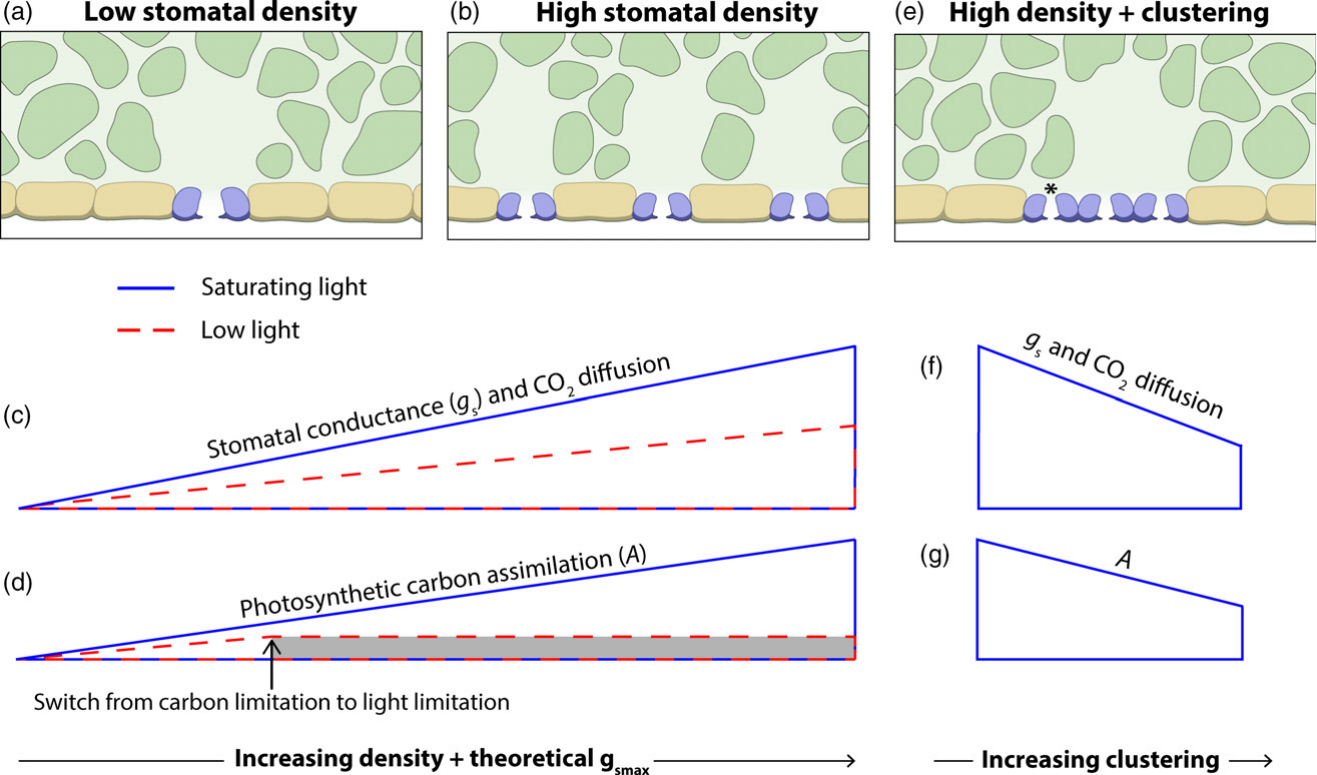
The generalised interactions between light intensity, theoretical stomatal conductance and gas exchange, in plants with altered stomatal density and patterning. Stylised cross sections of plants with (a) low stomatal density and (b) high stomatal density. Increasing stomatal density (*D*) leads to a corresponding increase in the theoretical maximum stomatal conductance (*g*_smax_). Assuming no compensatory mechanisms, the interactions between theoretical *g*_smax_, light intensity and gas exchange have been generalised as follows. (c) Increasing *D* and theoretical *g*_smax_ corresponds to an increase in stomatal conductance (*g*_s_) and the rate of CO_2_ diffusion to the chloroplast. The size and strength of this relationship is stronger under saturating light (blue line) compared with low light (red dashed line). (d) Under saturating light, increasing *D* and theoretical *g*_smax_, and thus *g*_s_ and CO_2_ diffusion, leads to an increase in carbon assimilation (*A*). However, under low light conditions, this benefit is only realised when carbon availability is limiting *A*. Once light becomes the limiting factor, as denoted by the greyed area, any increase to *D* and theoretical *g*_smax_ no longer increases *A*. The point at which *A* changes from being carbon limited to light limited is denoted by the arrow; however, this is dependent on species and environmental conditions. (e) An increase in *D* is often accompanied by an increase in stomatal clustering, as depicted in the stylised cross section. When stomata are present in clusters, the relationship between theoretical *g*_smax_ and gas exchange parameters, seen in (c) and (d), is weakened or broken. Whilst maintaining a high *D*, and thus high theoretical *g*_smax_, increasing stomatal clustering results in a decrease in (f) *g*_s_ and CO_2_ diffusion and (g) *A*, under saturating light. This may be explained by factors including altered diffusion kinetics and/or the incorrect alignment of stomata over mesophyll cells rather than substomatal cavities, as shown by the asterisk in (e).

Changes to *D* allows for the long‐term optimisation of a plant's gas exchange capacity such that, as growth conditions change, *C*_c_ does not limit *A*. For example, increases in *C*_a_, driven by the onset of the industrial revolution, have occurred in parallel with a general decrease in *D* (Woodward, 1987). This is believed to be due to higher *C*_a_ increasing the concentration gradient between *C*_a_ and *C*_i_, allowing plants to adjust their development to achieve optimum *A* with fewer stomata. Under high light conditions, in which there is an ample supply of ATP and NADPH produced by the light reactions of photosynthesis, *D* generally increases (Schoch *et al.*, 1980; Lake *et al.*, 2001). This augments the availability of CO_2_ at the site of carboxylation and promotes *A*. The relationship between environmental variables and the regulation of *D* has historically made it challenging to dissect out the relative importance of changes to *D* on the rate of *A*. However, over the last decade, a number of useful genetic resources have become available allowing direct modifications to the stomatal development pathway [see Zoulias *et al.* (2018)]. This has enabled in‐depth studies of the effects of manipulating *D* on plant physiology, without changing the natural growth environment of the plant (Doheny‐Adams *et al.*, 2012; Dow *et al.*, 2014b; Hepworth *et al.*, 2015; Hepworth *et al.*, 2016; Wang *et al.*, 2016). In particular, the discovery of the EPF family of secreted peptides has led to the engineering of plants with greatly altered *D* irrespective of light or *C*_a_. Such plants with abnormally low or high *D* have become an invaluable toolkit to assess the influence of *D,* and thus theoretical *g*_smax_, on gas exchange. It is generally accepted that a positive relationship between *D*, *g*_s_ and CO_2_ diffusion exists (Figure 2a–c); however, the effect of *D* on *A* is more complicated and is discussed below.

### Reductions in stomatal density

Reducing *D* has proved to be an effective method of enhancing drought tolerance and *iWUE* through reductions in plant water loss [recently reviewed by Bertolino *et al.* (2019) and Leakey *et al.* (2019)]. In theory, a reduction in *D* should lead to a decrease in both *g*_s_ and *A*. However, several studies have shown that in practice, this is not always the case. Research by Doheny‐Adams *et al.* (2012) highlighted how the manipulation of the EPF gene family could generate Arabidopsis plants with *D* ranging from 20 to 325% that of wild‐type. Gas exchange analysis on a subset of these plants showed that those with approximately 80% fewer stomata than wild‐type demonstrated significant reductions in both *g*_s_ (*c.* 50%) and *A* (*c.* 25%) under steady‐state conditions (Franks *et al.*, 2015). This disconnect between the magnitude of water loss and carbon gain resulted in substantial improvements to *iWUE*. In addition, no differences in estimations of photosynthetic biochemical efficiency were observed *in vivo* between genotypes, suggesting that the reductions in *A* resulted from lowered *g*_s_ and *C*_c_. As this study, translational research utilising cereal EPF orthologues has resulted in the reduction of *D* in several high yielding C_3_ crop species. Severe reductions in *D* of between 76 and 88% led to significant decreases in *A* in barley (*Hordeum vulgare*), rice (*Oryzae sativa*) and wheat (*Triticum aestivum*), under growth light conditions (Hughes *et al.*, 2017; Caine *et al.*, 2019; Dunn *et al.*, 2019). However, studies of rice and wheat lines that exhibited more moderate reductions in *D* of *c.* 46–58% showed no deleterious effect on *A.* Interestingly, in these cereal crop studies, the negative relationship between *S* and *D* did not hold true; smaller GCs were present in the barley and rice with severely decreased *D* (Hughes *et al.*, 2017; Caine *et al.*, 2019).

Together, these studies (Franks *et al.*, 2015; Hughes *et al.*, 2017; Caine *et al.*, 2019) show that plants can be produced that have approximately half the normal number of stomata with no detrimental effects on *A*, suggesting that a threshold exists before which reductions in *D* begin to affect *A.* The reason(s) for such a threshold in plants with manipulated EPF levels is currently unknown; however, there are several probable explanations. Firstly, at current ambient *C*_a_ levels, light intensity rather than *C*_c_ is more likely to limit *A*. In such circumstances, reducing *D* would not limit *A* until *C*_c_ becomes more limiting to *A* than the products of the light reaction (Figure 2d). Secondly, plants may compensate for reductions in *D* by altering leaf architecture in a manner that enhances CO_2_ diffusion to the chloroplast. Indeed, a level of coordination exists between the stomata and the underlying tissues, which affects both mesophyll cell and intercellular air characteristics (Dow *et al.*, 2017; Lundgren *et al.*, 2019). Finally, if a source of resistance within the CO_2_ diffusion pathway is greater than the increased stomatal resistance generated by the reduction in *D*, then this may place a bottleneck on CO_2_ movement to the site of carboxylation, and produce a greater limitation than that imposed by moderate reductions in *D*. This resistance is perhaps most likely to occur between the intercellular airspace and the chloroplast stroma (i.e. mesophyll resistance, see Figure 1b). However, estimations of *g*_m_ have yet to be conducted on plants with drastically reduced numbers of stomata. Regardless of the exact mechanism(s), it is clear that we can decouple *D* from *A*, and as a result, increase *iWUE* and improve yields under drought conditions (Wang *et al.*, 2016; Hughes *et al.*, 2017; Caine *et al.*, 2019; Dunn *et al.*, 2019; Mohammed *et al.*, 2019).

### Increases in stomatal density

Significant increases in *D* have most commonly been observed in studies which substantially alter *C*_a_ or light intensity (Casson and Gray, 2008). In terms of quantifying photosynthesis, this presents several confounding factors that make it difficult to dissect out the effects of increasing *D* from those of the altered growth environment. As a result, only a small number of studies are available for discussion. Fortunately, the same family of stomatal development regulators, the EPFs, can be exploited genetically to either reduce or increase *D.* Work by Tanaka *et al.* (2013) reported that Arabidopsis EPF mutants with *c.* 75% more stomata than wild‐type counterparts demonstrated significantly higher rates of *A* under high light intensities. This enhancement in *A* was thought to be driven by improvements to CO_2_ diffusion rather than alterations to the photosynthetic machinery or biochemistry. Interestingly, the same study also observed that increased *D* improved *A* under elevated *C*_a_. Under such conditions, RuBisCO should be saturated with CO_2_ regardless of increased *D,* as the concentration gradient between *C*_a_ and *C*_i_ is greater. The authors attributed this enhancement to improvements in the homogeneity of *C*_i_ throughout the leaf and thus lateral diffusion of CO_2_ (Morison and Lawson, 2007)*.* However, alterations to the mesophyll layer architecture facilitated by the presence of significantly more stomata cannot be overlooked, and have been observed in plants with manipulated EPF levels (Dow *et al.*, 2017; Lundgren *et al.*, 2019). Similar transgenic work in Arabidopsis utilising a different member of the EPF gene family produced plants with *c.* 130% more stomata than wild‐type (Franks *et al.*, 2015). In these plants, in agreement with the general trend highlighted in Figure 2(b,c), an increase in *D* caused a significant increase in both *g*_s_ and *C*_i_ under high light glasshouse conditions. However, despite a positive trend, no significant difference in *A* was reported under these conditions, unlike Tanaka *et al.* (2013), suggesting possible limitations to *A* beyond gas exchange.

Some studies have increased *D* without the direct manipulation of EPF gene expression. For example, transgenic work in Arabidopsis manipulating a negative regulator of stomatal development referred to as STOMATAL DENSITY AND DISTRIBUTION 1 (SDD‐1) generated plants with a 2.5 fold increase in *D* (Schlüter *et al.*, 2003). No increase in *A* was observed in these plants under growth light conditions of 200–250 µmol m^−2^ sec^−1^. However, upon exposure to high light, these transgenic plants exhibited elevated levels of *A* in comparison with wild‐type. These data suggest that, under moderate growth light, products of the light reaction rather than *C*_c_ restrict maximum levels of *A* (see greyed area of Figure 2d). Upon transfer to higher light intensities, electron transport is likely to have increased in all plants. However, the presence of additional stomata in *sdd‐1* plants may promote increased *C*_c_ and have facilitated an increase in *A*. In addition, the manipulation of the SHORTROOT developmental pathway in rice has generated transgenic lines with approximately 40% more stomata than controls, and a *c.* 20–40% increase in theoretical *g*_smax_ (Schuler *et al.*, 2018). Interestingly, however, when the response to changing light intensity was measured (transition from 100 to 1000 µmol m^−2^ sec^−1^), no significant increases in either *g*_s_ or *A* were observed. This suggests that under some circumstances plants may compensate for increased *D* by reducing their operating *g*_s,_ via alterations in their stomatal apertures, in an effort to limit the amount of water lost through *E*.

### Stomatal patterning

The patterning of stomatal complexes across the leaf epidermis differs markedly within the plant kingdom, and in particular, between the two major flowering plant groups (Rudall and Knowles, 2013). In broad‐leaved eudicots, stomata are found scattered throughout the epidermis in a seemingly irregular fashion. This is in contrast to grasses, whose stomata develop in parallel rows within pre‐defined epidermal cell files. This variation in stomatal patterning is a product of differences in the temporal and spatial organisation of leaf cell division and cell expansion that takes place in these two groups of angiosperms (Nelissen *et al.*, 2016). Despite fundamental differences in leaf growth characteristics, the one‐cell spacing rule is upheld, utilising the same genetic toolbox – albeit ‘alternatively wired’ in the grasses (Raissig *et al.*, 2016). It has long been presumed that adequate spacing is required for proper stomatal function, as GCs require ionic exchange with neighbouring cells in order to alter stomatal aperture (Outlaw, 1983; Kim *et al.*, 2010). In addition, GC function may be promoted by mechanical forces produced by the turgor pressure of surrounding cells (Edwards *et al.*, 1976). Thus, directly adjacent stomatal complexes may end up competing with each other to generate or release turgor.

Through the manipulation of genetic components responsible for regulating stomatal spacing, a range of transgenic Arabidopsis genotypes have been generated, which exhibit clusters of stomata in direct contact with each other (contiguous) overlaying a shared substomatal cavity (Figure 2e). This has allowed the importance of the one‐cell spacing rule on stomatal function and photosynthetic productivity to be investigated. By comparing high and low clustering genotypes that have similar *S* and *D*, Dow *et al.* (2014b) found that measured *g*_smax_ was consistently reduced in lines with highly clustered stomata (Figure 2f). Closure of stomata located in clusters was also reported to be slower in response to changes in environmental conditions. It has been proposed that the suppressed dynamic range and kinetics of contiguous stomata might be due to mechanical failure of the GCs. This may occur through the lack of neighbouring epidermal cells, which limits the supply of necessary ion reserves and/or the physical interaction that facilitates GC movement. Work by Papanatsiou *et al.* (2016) confirmed that the GCs of *tmm1* mutants, with high *D* and clustering, have reduced K^+^ accumulation and K^+^ channel activities. However, as these experiments were performed on detached epidermal peels in unlimited K^+^ concentrations, they argue that stomatal clustering causes fundamental changes in GC ion transport that goes over and above the explanation of inadequate ion exchange discussed above. Stomatal clustering led to a significant reduction in *A* (Figure 2g) when compared with uniformly distributed stomata of similar *D* (Dow *et al.*, 2014b), likely due to their lower operating *g*_s_. Interestingly, *A* in *tmm1* plants was also significantly reduced compared with wild‐type controls under high light, despite having considerably more stomata and a comparable operating *g*_s_ (Papanatsiou *et al.*, 2016). This may be explained by a misalignment between the stomata, intercellular airspaces and underlying photosynthetic tissue in the transgenic plants (see asterisk in Figure 2e), as suggested by Dow *et al.* (2014b). The coordination that exists between the formation of stomata and their substomatal cavities (Lundgren *et al.*, 2019) may have been disrupted when altering the developmental signalling responsible for enforcing cell spacing. This may impede CO_2_ diffusion through the mesophyll, leading to an uneven *C*_i_ and reduced photosynthetic potential of the leaf. Furthermore, the reduced diffusive capacity of clustered stomata may also be due to their close proximity with each other and their shared substomatal cavity. Previous models have suggested that the overlapping vapour shells from clustered stomata can reduce *E* by 5–15% (Lehmann and Or, 2015). Whilst the effects of multiple stomata overlaying the same substomatal cavity has not been directly explored, models have demonstrated that substomatal cavity size may influence gaseous exchange. For example, it was reported that a substomatal cavity should be at least double the width of the stomatal pore in order to minimise water loss (Pickard, 1981), and whilst increasing it beyond this was found to offer no further decrease in *E*, it continued to promote CO_2_ (Pickard, 1982; Roth‐Nebelsick, 2007). This was achieved through an increase in the surface area of the intercellular airspace in contact with assimilating mesophyll tissue, which in turn increased the CO_2_ sink (Roth‐Nebelsick, 2007). In the case of clustered stomata, the size ratio between the substomatal cavity and the total combined pore area may be reduced, which could result in a decrease in the internal CO_2_ gradient of the cavity and thus *A.* As a result of the factors discussed above, the relationship seen between theoretical *g*_smax_ and both operating *g*_s_ and *A* (Figure 2c,d) is broken (Figure 2f,g).

Whilst one‐cell stomatal spacing is evident in the majority of plant species, there are several species that have deviated from the rule; such as members of the Begonia genus, which show stomatal clustering when growing in their natural habitat (Gan *et al*., 2010). For example, *Begonia plebeja* forms clusters of two or more non‐contiguous stomata that are separated by an abnormally narrow non‐stomatal epidermal cell and share a substomatal cavity (Neubauer, 1967; Burt‐Utley and Utley, 1999). In addition, this species displays increased numbers of smaller stomata in comparison with other *Begonia* species that exhibit normally spaced stomata (Papanatsiou *et al.*, 2017). Whilst individual pore apertures of *B. plebeja* stomata are reduced, measured *g*_smax_ was significantly greater, due to the higher *D* (Papanatsiou *et al.*, 2017). Despite this, *B. plebeja* show lower rates of *A* under saturating light in comparison with non‐clustered *Begonia* species. These results support the transgenic studies discussed above which show that stomatal clustering negatively impacts on CO_2_ uptake, and thus *A*, particularly in conditions in which light is not limiting. However, in contrast to the above studies, the clustered stomata of *B. plebeja* were reported to close faster, aided by their smaller *S*. The presence of intervening pavement cells, although small, may alleviate the restrictions imposed on GC movement by adjoining stomata. Under low light conditions, *iWUE* was in fact improved by 30%, suggesting that non‐contiguous clustering of small stomata could provide an adaptive strategy in water restricted and/or low light environments (Papanatsiou *et al.*, 2017). Indeed, several clustered *Begonia *species are found growing on rocks near waterfalls which experience low evaporative demand, and in shaded areas underneath the forest canopy (Hoover, 1986). As a whole, these results indicate that stomatal clustering impacts negatively on *A*, except in specific examples in which it can offer an advantage, as explained above.

The distribution of stomata between the upper (adaxial) and lower (abaxial) leaf surfaces, known as stomatal ratio (*R*), may also affect leaf function and plant productivity (Jordan *et al.*, 2014). An often overlooked aspect of stomatal studies is that the majority of plant species are hypostomatous, in which stomata are solely restricted to the abaxial surface of their leaves (*R* = 0). Whilst far less common in nature, some species are amphistomatous, with stomata found equally distributed between both leaf surfaces (*R* = 0.5; Muir, 2015). Although these two types are most predominantly seen, intermediates in *R* are known to exist. Stomatal development is believed to be differentially controlled between the two leaf surfaces (Lake *et al.*, 2002) and certain species show a high degree of plasticity in *R* in response to changes in their environment (Mott and Michaelson, 1991; James and Bell, 2000)*.* Amphistomy generally occurs in high light environments, in either fast growing herbaceous crops (Metcalfe and Chalk, 1950) or slow‐growing arid species (Parkhurst, 1978), and is believed to offer an evolutionary advantage in such conditions. By doubling the surface area for gas exchange to take place, this reduces boundary layer resistance and facilitates greater CO_2_ uptake for photosynthesis (Parkhurst, 1978). Amphistomy also shortens the pathway for CO_2_ transport between the atmosphere and the mesophyll chloroplasts, which is particularly beneficial in thick leaves: a common trait in high light (Parkhurst and Mott, 1990; Drake *et al.*, 2019). Furthermore, it has been suggested that amphistomy may reduce temperature gradients and prevent the condensation of water within the leaf, which could limit the diffusion of CO_2_ (Buckley *et al.*, 2017). However, increased *A* also comes at a cost of increased *E*. This is especially problematic for dorsiventral leaves, in which sunlight hitting the upper leaf surface causes the adaxial stomata to experience greater irradiance, temperature and evaporative demand (Rockwell *et al.*, 2014), making the most photosynthetically active tissue prone to harmfully fast desiccation. To prevent this from happening, amphistomatous leaves are required to increase their hydraulic capacity through additional investment in vascular tissue (Drake *et al.*, 2019). It is also important for stomata to be independently controlled on both leaf surfaces to optimise stomatal apertures to the conditions that each surface is experiencing. Studies have shown that the abaxial and adaxial stomata of several species are indeed able to respond autonomously to changing conditions (Lu, 1989; Lu *et al.*, 1993; Richardson *et al.*, 2017) however this is not always the case (Mott and Peak, 2018). Using a modelling approach, Muir (2019) found that the increased *A* almost always outweighs the risk of increased water loss. Therefore some other cost, which warrants further investigation, is yet to be identified to explain the rarity of amphistomy in the plant kingdom. Despite the potential benefits of amphistomy on photosynthetic gas exchange, it has been largely ignored by the field. By optimising stomatal ratio, this may allow plants to utilise high light more efficiently, and should be considered, alongside other stomatal traits, for future bioengineering approaches towards improving photosynthesis.

## Concluding remarks

Stomata are an essential part of the photosynthetic machinery, and it is common for studies that examine plants with altered photosynthetic performance to also report differences in stomatal behaviour or *g*_s_ (Lawson *et al.*, 2008; Simkin *et al.*, 2015; Cui *et al.*, 2016; Głowacka *et al.*, 2018). This highlights the fact that, although often studied independently, the stomatal uptake of CO_2_ and the dark and light reactions of photosynthesis are intimately coupled. Furthermore, research into the functional relationship between stomata and *A* is revealing a more complex picture than previously thought, with a coordinated response between changes to *g*_s_ and the rate of *A* not backed up by experimental data. This complexity may arise due to distinct limitations to *A* occurring under certain environmental conditions, as outlined in Figure 2(a–d). In summary, only when *A* is limited by the supply of CO_2_ would an increase in *g*_s_ bring about an advantage. Conversely, only when *A* is biochemically limited by NADPH and ATP concentrations or RuBP regeneration would a reduction in *g*_s_ not be deleterious to *A*.

It is evident from the contrasting results discussed in this review, that there are significant gaps in our understanding of the precise functional coordination between *D* and *A*. In terms of reducing *D*, future work should aim to measure *A* under a broader range of steady‐state light intensities. This work would allow us to understand at what point CO_2_ diffusion becomes more limiting than the processes of the light reactions. Such results may be species‐specific, with some species more capable of maintaining optimum levels of *A* when possessing reduced numbers of stomata than others. When increasing *D*, future work should seek to understand how we may decouple compensatory mechanisms, such as a reduction in stomatal aperture which may result in an unchanged *g*_s_ [as noted in Schuler *et al.* (2018) and Mohammed *et al.* (2019)]. Consideration should also be given towards the optimisation of stomatal ratio between the two leaf surfaces, and what, if any, physiological relevance this may have on gas exchange. Furthermore, it would be interesting to assess how plants with altered stomatal morphology or distribution perform under fluctuating light conditions, as the transitions between carbon and light limiting photosynthesis would occur more rapidly and frequently. Under these conditions, perhaps the engineering of ‘speedy stomata’ through altered GC size, shape or biochemistry would be more beneficial to improving *A* than changes to *D* (Raven, 2014). For example, recent work by Papanatsiou *et al.* (2019) incorporated a synthetic ion channel into Arabidopsis GCs. By doing so, both stomatal dynamics and plant growth were improved, especially under fluctuating light conditions. Finally, improvements to photosynthesis through the generation of plants with increased *D* or *g*_s_ would potentially benefit from accompanying improvements to the efficiency of the electron transport chain or enhanced flux through the Calvin‐Benson cycle.

## Author contributions

The authors contributed equally to the writing of this review.

## Conflict of interest

The authors declare no conflict of interest.

## References

[tpj14560-bib-0001] Assmann, S.M. and Zeiger, E. (1987) Guard cell bioenergetics In Stomatal Function (Zeiger E., Farquhar G. and Cowan I., eds). Stanford, CA: Stanford Univ. Press, pp. 163–194.

[tpj14560-bib-0002] Bertolino, L.T. , Caine, R.S. and Gray, J.E. (2019) Impact of stomatal density and morphology on water‐sse efficiency in a changing world. Front. Plant Sci. 10, 225. 30894867 10.3389/fpls.2019.00225 PMC6414756

[tpj14560-bib-0003] de Boer, H.J. , Price, C.A. , Wagner‐Cremer, F. , Dekker, S.C. , Franks, P.J. and Veneklaas, E.J. (2016) Optimal allocation of leaf epidermal area for gas exchange. New Phytol. 210, 1219–1228. 26991124 10.1111/nph.13929 PMC5069575

[tpj14560-bib-0004] Buckley, T.N. , John, G.P. , Scoffoni, C. and Sack, L. (2017) The sites of evaporation within leaves. Plant Physiol. 173, 1763–1782. 28153921 10.1104/pp.16.01605 PMC5338672

[tpj14560-bib-0005] Burt‐Utley, K. and Utley, J.F. (1999) Contributions toward a revision of Begonia section Weilbachia (Begoniaceae). Novon, 9, 483–489.

[tpj14560-bib-0006] Caemmerer, S. and Evans, J.R. (1991) Determination of the average partial pressure of CO_2_ in chloroplasts from leaves of several C3 plants. Funct. Plant Biol. 18, 287–305.

[tpj14560-bib-0007] Caine, R.S. , Yin, X. , Sloan, J. ***et al*** **.** (2019) Rice with reduced stomatal density conserves water and has improved drought tolerance under future climate conditions. New Phytol. 221, 371–384. 30043395 10.1111/nph.15344 PMC6492113

[tpj14560-bib-0008] Carter, R. , Woolfenden, H. , Baillie, A. , Amsbury, S. , Carroll, S. , Healicon, E. , Sovatzoglou, S. , Braybrook, S. , Gray, J.E. and Hobbs, J. (2017) Stomatal opening involves polar, not radial, stiffening of guard cells. Curr. Biol. 27, 2974–2983. e2972. 28943087 10.1016/j.cub.2017.08.006 PMC5640513

[tpj14560-bib-0009] Casson, S. and Gray, J.E. (2008) Influence of environmental factors on stomatal development. New Phytol. 178, 9–23. 18266617 10.1111/j.1469-8137.2007.02351.x

[tpj14560-bib-0010] Chater, C.C. , Caine, R.S. , Tomek, M. , ***et al.*** (2016) Origin and function of stomata in the moss Physcomitrella patens. Nat. Plants, 2, 16179. 27892923 10.1038/nplants.2016.179 PMC5131878

[tpj14560-bib-0011] Chen, Z.‐H. , Chen, G. , Dai, F. , Wang, Y. , Hills, A. , Ruan, Y.‐L. , Zhang, G. , Franks, P.J. , Nevo, E. and Blatt, M.R. (2017) Molecular evolution of grass stomata. Trends Plant Sci. 22, 124–139. 27776931 10.1016/j.tplants.2016.09.005

[tpj14560-bib-0012] Crowley, T.J. and Berner, R.A. (2001) CO_2_ and climate change. Science (New York, N.Y.), 292, 870–872. 10.1126/science.1061664 11341284

[tpj14560-bib-0013] Cui, L.‐L. , Lu, Y.‐S. , Li, Y. , Yang, C. and Peng, X.‐X. (2016) Overexpression of glycolate oxidase confers improved photosynthesis under high light and high temperature in rice. Front. Plant Sci. 7, 1165 10.3389/fpls.2016.01165 27540387 PMC4972838

[tpj14560-bib-0014] Dilcher, D. (2000) Toward a new synthesis: major evolutionary trends in the angiosperm fossil record. Proc. Natl Acad. Sci. USA, 97, 7030–7036. 10860967 10.1073/pnas.97.13.7030 PMC34380

[tpj14560-bib-0015] Doheny‐Adams, T. , Hunt, L. , Franks, P.J. , Beerling, D.J. and Gray, J.E. (2012) Genetic manipulation of stomatal density influences stomatal size, plant growth and tolerance to restricted water supply across a growth carbon dioxide gradient. Philos. Trans. Royal Soc. Lond. B Biol. Sci. 367, 547–555. 10.1098/rstb.2011.0272 PMC3248714 22232766

[tpj14560-bib-0016] Dow, G.J. , Bergmann, D.C. and Berry, J.A. (2014a) An integrated model of stomatal development and leaf physiology. New Phytol. 201, 1218–1226. 24251982 10.1111/nph.12608

[tpj14560-bib-0017] Dow, G.J. , Berry, J.A. and Bergmann, D.C. (2014b) The physiological importance of developmental mechanisms that enforce proper stomatal spacing in Arabidopsis thaliana. New Phytol. 201, 1205–1217. 24206523 10.1111/nph.12586

[tpj14560-bib-0018] Dow, G.J. , Berry, J.A. and Bergmann, D.C.J.N.P. (2017) Disruption of stomatal lineage signaling or transcriptional regulators has differential effects on mesophyll development, but maintains coordination of gas exchange. New Phytol. 216, 69–75. 28833173 10.1111/nph.14746 PMC5601202

[tpj14560-bib-0019] Drake, P.L. , Froend, R.H. and Franks, P.J. (2013) Smaller, faster stomata: scaling of stomatal size, rate of response, and stomatal conductance. J. Exp. Bot. 64, 495–505. 23264516 10.1093/jxb/ers347 PMC3542046

[tpj14560-bib-0020] Drake, P.L. , de Boer, H.J. , Schymanski, S.J. and Veneklaas, E.J. (2019) Two sides to every leaf: water and CO_2_ transport in hypostomatous and amphistomatous leaves. New Phytol. 222, 1179–1187. 30570766 10.1111/nph.15652

[tpj14560-bib-0021] Dunn, J. , Hunt, L. , Afsharinafar, M. , Meselmani, M.A. , Mitchell, A. , Howells, R. , Wallington, E. , Fleming, A.J. and Gray, J.E. (2019) Reduced stomatal density in bread wheat leads to increased water‐use efficiency. J. Exp. Bot. 70, 4737–4748. 31172183 10.1093/jxb/erz248 PMC6760291

[tpj14560-bib-0022] Edwards, M. , Meidner, H. and Sheriff, D. (1976) Direct measurements of turgor pressure potentials of guard cells: II. The mechanical advantage of subsidiary cells, The spannunqsphase, and the optimum leaf water deficit. J. Exp. Bot. 27, 163–171.

[tpj14560-bib-0023] Elliott‐Kingston, C. , Haworth, M. , Yearsley, J.M. , Batke, S.P. , Lawson, T. and McElwain, J.C. (2016) Does size matter? Atmospheric CO_2_ may be a stronger driver of stomatal closing rate than stomatal size in taxa that diversified under low CO_2_ . Front. Plant Sci. 7, 1253. 27605929 10.3389/fpls.2016.01253 PMC4996050

[tpj14560-bib-0024] Evans, J.R. and Von Caemmerer, S. (1996) Carbon dioxide diffusion inside leaves. Plant Physiol. 110, 339. 12226185 10.1104/pp.110.2.339 PMC157726

[tpj14560-bib-0025] Evans, J.R. , Kaldenhoff, R. , Genty, B. and Terashima, I. (2009) Resistances along the CO_2_ diffusion pathway inside leaves. J. Exp. Bot. 60, 2235–2248. 19395390 10.1093/jxb/erp117

[tpj14560-bib-0026] Farquhar, G.D. , von Caemmerer, S. and Berry, J.A. (1980) A biochemical model of photosynthetic CO_2_ assimilation in leaves of C3 species. Planta, 149, 78–90. 24306196 10.1007/BF00386231

[tpj14560-bib-0027] Franks, P.J. (2006) Higher rates of leaf gas exchange are associated with higher leaf hydrodynamic pressure gradients. Plant Cell Environ. 29, 584–592. 17080609 10.1111/j.1365-3040.2005.01434.x

[tpj14560-bib-0028] Franks, P.J. and Beerling, D.J. (2009) Maximum leaf conductance driven by CO_2_ effects on stomatal size and density over geologic time. Proc. Natl Acad. Sci. USA, 106, 10343–10347. 19506250 10.1073/pnas.0904209106 PMC2693183

[tpj14560-bib-0029] Franks, P.J. and Farquhar, G.D. (2007) The mechanical diversity of stomata and its significance in gas‐exchange control. Plant Physiol. 143, 78–87. 17114276 10.1104/pp.106.089367 PMC1761988

[tpj14560-bib-0030] Franks, P.J. , Leitch, I.J. , Ruszala, E.M. , Hetherington, A.M. and Beerling, D.J. (2012) Physiological framework for adaptation of stomata to CO_2_ from glacial to future concentrations. Philos. Trans. Royal Soc. B Biol. Sci. 367, 537–546. 10.1098/rstb.2011.0270 PMC3248712 22232765

[tpj14560-bib-0031] Franks, P.J. , W. Doheny‐Adams, T. , Britton‐Harper, Z.J. and Gray, J.E. (2015) Increasing water‐use efficiency directly through genetic manipulation of stomatal density. New Phytol. 207, 188–195. 25754246 10.1111/nph.13347

[tpj14560-bib-0032] Gan, Y. , Zhou, L. , Shen, Z.-J. , Shen, Z.-X. , Zhang, Y.-Q. and Wang, G.-X. (2010) Stomatal clustering, a new marker for environmental perception and adaptation in terrestrial plants. Bot. Stud. 51, 325–336.

[tpj14560-bib-0033] Geisler, M. , Nadeau, J. and Sack, F.D. (2000) Oriented asymmetric divisions that generate the stomatal spacing pattern in Arabidopsis are disrupted by the too many mouths mutation. Plant Cell, 12, 2075–2086. 11090210 10.1105/tpc.12.11.2075 PMC150159

[tpj14560-bib-0034] Głowacka, K. , Kromdijk, J. , Kucera, K. , Xie, J. , Cavanagh, A.P. , Leonelli, L. , Leakey, A.D.B. , Ort, D.R. , Niyogi, K.K. and Long, S.P. (2018) Photosystem II Subunit S overexpression increases the efficiency of water use in a field‐grown crop. Nat. Commun. 9, 868. 29511193 10.1038/s41467-018-03231-x PMC5840416

[tpj14560-bib-0035] Haworth, M. , Scutt, C.P. , Douthe, C. , Marino, G. , Gomes, M.T.G. , Loreto, F. , Flexas, J. and Centritto, M. (2018) Allocation of the epidermis to stomata relates to stomatal physiological control: stomatal factors involved in the evolutionary diversification of the angiosperms and development of amphistomaty. Environ. Exp. Bot. 151, 55–63.

[tpj14560-bib-0036] Hepworth, C. , Doheny‐Adams, T. , Hunt, L. , Cameron, D.D. and Gray, J.E. (2015) Manipulating stomatal density enhances drought tolerance without deleterious effect on nutrient uptake. New Phytol. 208, 336–341. 26268722 10.1111/nph.13598 PMC4973681

[tpj14560-bib-0037] Hepworth, C. , Turner, C. , Landim, M.G. , Cameron, D. and Gray, J.E. (2016) Balancing water uptake and loss through the coordinated regulation of stomatal and root development. PLoS ONE, 11, e0156930. 27275842 10.1371/journal.pone.0156930 PMC4898744

[tpj14560-bib-0038] Hepworth, C. , Caine, R.S. , Harrison, E.L. , Sloan, J. and Gray, J.E. (2018) Stomatal development: focusing on the grasses. Curr. Opin. Plant Biol. 41, 1–7. 28826033 10.1016/j.pbi.2017.07.009

[tpj14560-bib-0039] Hetherington, A.M. and Woodward, F.I. (2003) The role of stomata in sensing and driving environmental change. Nature, 424, 901–908. 12931178 10.1038/nature01843

[tpj14560-bib-0040] Hoover, W.S. (1986) Stomata and stomatal clusters in begonia: ecological response in two Mexican species. Biotropica, 18, 16–21.

[tpj14560-bib-0041] Hughes, J. , Hepworth, C. , Dutton, C. , Dunn, J.A. , Hunt, L. , Stephens, J. , Cameron, D. , Waugh, R. and Gray, J.E. (2017) Reducing stomatal density in barley improves drought tolerance without impacting on yield. Plant Physiol. 174, 776–787. 28461401 10.1104/pp.16.01844 PMC5462017

[tpj14560-bib-0042] James, S.A. and Bell, D.T. (2000) Influence of light availability on leaf structure and growth of two Eucalyptus globulus ssp. globulus provenances. Tree Physiol. 20, 1007–1018. 11305455 10.1093/treephys/20.15.1007

[tpj14560-bib-0043] Jordan, G.J. , Carpenter, R.J. and Brodribb, T.J. (2014) Using fossil leaves as evidence for open vegetation. Palaeogeogr. Palaeoclimatol. Palaeoecol. 395, 168–175.

[tpj14560-bib-0044] Kim, T.H. , Bohmer, M. , Hu, H. , Nishimura, N. and Schroeder, J.I. (2010) Guard cell signal transduction network: advances in understanding abscisic acid, CO_2_, and Ca^2+^ signaling. Annu. Rev. Plant Biol. 61, 561–591. 20192751 10.1146/annurev-arplant-042809-112226 PMC3056615

[tpj14560-bib-0045] Kollist, H. , Nuhkat, M. and Roelfsema, M.R.G. (2014) Closing gaps: linking elements that control stomatal movement. New Phytol. 203, 44–62. 24800691 10.1111/nph.12832

[tpj14560-bib-0046] Lake, J.A. , Quick, W.P. , Beerling, D.J. and Woodward, F.I. (2001) Plant development: Signals from mature to new leaves. Nature, 411, 154–154. 11346781 10.1038/35075660

[tpj14560-bib-0047] Lake, J.A. , Woodward, F.I. and Quick, W.P. (2002) Long‐distance CO_2_ signalling in plants. J. Exp. Bot. 53, 183–193. 11807121 10.1093/jexbot/53.367.183

[tpj14560-bib-0048] Lawson, T. and Blatt, M. (2014) Stomatal size, speed and responsiveness impact on photosynthesis and water use efficiency. Plant Physiol. 164, 1556–1570. 24578506 10.1104/pp.114.237107 PMC3982722

[tpj14560-bib-0049] Lawson, T. and Vialet‐Chabrand, S. (2019) Speedy stomata, photosynthesis and plant water use efficiency. New Phytol. 221, 93–98. 29987878 10.1111/nph.15330

[tpj14560-bib-0050] Lawson, T. , Lefebvre, S. , Baker, N.R. , Morison, J.I.L. and Raines, C.A. (2008) Reductions in mesophyll and guard cell photosynthesis impact on the control of stomatal responses to light and CO_2_ . J. Exp. Bot. 59, 3609–3619. 18836187 10.1093/jxb/ern211 PMC2561148

[tpj14560-bib-0051] Leakey, A.D.B. , Ferguson, J.N. , Pignon, C.P. , Wu, A. , Jin, Z. , Hammer, G.L. and Lobell, D.B. (2019) Water use efficiency as a constraint and target for improving the resilience and productivity of C3 and C4 crops. Annu. Rev. Plant Biol. 70, 781–808. 31035829 10.1146/annurev-arplant-042817-040305

[tpj14560-bib-0052] Lehmann, P. and Or, D. (2015) Effects of stomata clustering on leaf gas exchange. New Phytol. 207, 1015–1025. 25967110 10.1111/nph.13442

[tpj14560-bib-0053] Liu, T. , Ohashi‐Ito, K. and Bergmann, D.C. (2009) Orthologs of Arabidopsis thaliana stomatal bHLH genes and regulation of stomatal development in grasses. Development, 136, 2265–2276. 19502487 10.1242/dev.032938

[tpj14560-bib-0054] Lloyd, J. , Syvertsen, J.P. , Kriedemann, P.E. and Farquhar, G.D. (1992) Low conductances for CO_2_ diffusion from stomata to the sites of carboxylation in leaves of woody species. Plant Cell Environ. 15, 873–899.

[tpj14560-bib-0055] Lomax, B.H. , Woodward, F.I. , Leitch, I.J. , Knight, C.A. and Lake, J.A. (2009) Genome size as a predictor of guard cell length in Arabidopsis thaliana is independent of environmental conditions. New Phytol. 181, 311–314. 19054335 10.1111/j.1469-8137.2008.02700.x

[tpj14560-bib-0056] Lu, Z.‐M. (1989) Ratio of stomatal resistance on two sides of wheat leaves as affected by soil water content. Agric. For. Meteorol. 49, 1–7.

[tpj14560-bib-0057] Lu, Z. , Quinones, M. and Zeiger, E. (1993) Abaxial and adaxial stomata from Pima cotton (Gossypium barbadense L.) differ in their pigment content and sensitivity to light quality. Plant Cell Environ. 16, 851–858.

[tpj14560-bib-0058] Lundgren, M.R. , Mathers, A. , Baillie, A.L. ***et al*** **.** (2019) Mesophyll porosity is modulated by the presence of functional stomata. Nat. Commun. 10, 2825. 31249299 10.1038/s41467-019-10826-5 PMC6597550

[tpj14560-bib-0059] Matthews, J.S.A. , Vialet-Chabrand, S. and Lawson, T. (2018) Acclimation to fluctuating light impacts the rapidity of response and diurnal rhythm of stomatal conductance. Plant Physiol. 176, 1939–1951. 29371250 10.1104/pp.17.01809 PMC5841698

[tpj14560-bib-0060] McAusland, L. , Vialet‐Chabrand, S. , Davey, P. , Baker, N.R. , Brendel, O. and Lawson, T. (2016) Effects of kinetics of light‐induced stomatal responses on photosynthesis and water‐use efficiency. New Phytol. 211, 1209–1220. 27214387 10.1111/nph.14000 PMC4982059

[tpj14560-bib-0061] Metcalfe, C.R. and Chalk, L. (1950) Anatomy of the Dicotyledons: leaves, stem, and wood, in relation to taxonomy, with notes on economic uses. *Anatomy of the Dicotyledons: leaves, stem, and wood, in relation to taxonomy, with notes on economic uses*.

[tpj14560-bib-0062] Mishra, M.K. (1997) Stomatal characteristics at different ploidy levels in Coffea L. Ann. Bot. 80, 689–692.

[tpj14560-bib-0063] Mohammed, U. , Caine, R.S. , Atkinson, J.A. , Harrison, E.L. , Wells, D. , Chater, C.C. , Gray, J.E. , Swarup, R. and Murchie, E.H. (2019) Rice plants overexpressing OsEPF1 show reduced stomatal density and increased root cortical aerenchyma formation. Sci. Rep. 9, 5584. 30944383 10.1038/s41598-019-41922-7 PMC6447545

[tpj14560-bib-0064] Morison, J.I. and Lawson, T. (2007) Does lateral gas diffusion in leaves matter? Plant Cell Environ. 30, 1072–1085. 17661748 10.1111/j.1365-3040.2007.01685.x

[tpj14560-bib-0065] Mott, K.A. and Michaelson, O. (1991) Amphistomy as an adaptation to high light intensity in Ambrosia cordifolia (Compositae). Am. J. Bot. 78, 76–79.

[tpj14560-bib-0066] Mott, K.A. and Peak, D. (2018) Effects of the mesophyll on stomatal responses in amphistomatous leaves. Plant Cell Environ. 41, 2835–2843. 30073677 10.1111/pce.13411

[tpj14560-bib-0067] Muir, C.D. (2015) Making pore choices: repeated regime shifts in stomatal ratio. Proc. Royal Soc. B Biol. Sci. 282, 20151498. 10.1098/rspb.2015.1498 PMC4632635 26269502

[tpj14560-bib-0068] Muir, C.D. (2019) Is amphistomy an adaptation to high light? Optimality models of stomatal traits along light gradients. BioRxiv, 601377. 10.1093/icb/icz085 31141118

[tpj14560-bib-0069] Nelissen, H. , Gonzalez, N. and Inze, D. (2016) Leaf growth in dicots and monocots: so different yet so alike. Curr. Opin. Plant Biol. 33, 72–76. 27344391 10.1016/j.pbi.2016.06.009

[tpj14560-bib-0070] Neubauer, H.F. (1967) Bemerkungen uber den Bau der Begoniaceen In Berichte der Deutschen Botanischen Gesellschaft. Gustav Fischer Verlag, Jena, Germany, Vol. 80, pp. 80–97.

[tpj14560-bib-0071] Nunes, T.D.G. , Zhang, D. and Raissig, M.T. (2019) Form, development and function of grass stomata. Plant J. 101, 780–799. 31571301 10.1111/tpj.14552

[tpj14560-bib-0072] Ohsumi, A. , Kanemura, T. , Homma, K. , Horie, T. and Shiraiwa, T. (2007) Genotypic variation of stomatal conductance in relation to stomatal density and length in rice (Oryza sativa L.). Plant Prod. Sci. 10, 322–328.

[tpj14560-bib-0073] Outlaw, W. Jr (1983) Current concepts on the role of potassium in stomatal movements. Physiol. Plant. 59, 302–311.

[tpj14560-bib-0074] Papanatsiou, M. , Amtmann, A. and Blatt, M.R. (2016) Stomatal spacing safeguards stomatal dynamics by facilitating guard cell ion transport independent of the epidermal solute reservoir. Plant Physiol. 172, 254–263. 27406168 10.1104/pp.16.00850 PMC5074606

[tpj14560-bib-0075] Papanatsiou, M. , Amtmann, A. and Blatt, M.R. (2017) Stomatal clustering in Begonia associates with the kinetics of leaf gaseous exchange and influences water use efficiency. J. Exp. Bot. 68, 2309–2315. 28369641 10.1093/jxb/erx072 PMC5447881

[tpj14560-bib-0076] Papanatsiou, M. , Petersen, J. , Henderson, L. , Wang, Y. , Christie, J.M. and Blatt, M.R. (2019) Optogenetic manipulation of stomatal kinetics improves carbon assimilation, water use, and growth. Science (New York, N.Y.), 363, 1456–1459. 10.1126/science.aaw0046 30923223

[tpj14560-bib-0077] Parkhurst, D.F. (1978) The adaptive significance of stomatal occurrence on one or both surfaces of leaves. J. Ecol. 66, 367–383.

[tpj14560-bib-0078] Parkhurst, D.F. and Mott, K.A. (1990) Intercellular diffusion limits to CO_2_ uptake in leaves: studies in air and helox. Plant Physiol. 94, 1024–1032. 16667792 10.1104/pp.94.3.1024 PMC1077337

[tpj14560-bib-0079] Peterson, K.M. , Rychel, A.L. and Torii, K.U. (2010) Out of the mouths of plants: the molecular basis of the evolution and diversity of stomatal development. Plant Cell, 22, 296–306. 20179138 10.1105/tpc.109.072777 PMC2845417

[tpj14560-bib-0080] Pickard, W.F. (1981) How does the shape of the substomatal chamber affect transpirational water loss? Math. Biosci. 56, 111–127.

[tpj14560-bib-0081] Pickard, W. (1982) Distribution of evaporation in the sub‐stomatal chamber, the possibility of transpiration‐linked pore narrowing, and the pathway of water near the site of evaporation. Ann. Bot. 49, 545–548.

[tpj14560-bib-0082] Pillitteri, L.J. and Dong, J. (2013) Stomatal development in Arabidopsis. Arabidopsis Book, 11, e0162–e0162. 23864836 10.1199/tab.0162 PMC3711358

[tpj14560-bib-0083] Raissig, M.T. , Abrash, E. , Bettadapur, A. , Vogel, J.P. and Bergmann, D.C. (2016) Grasses use an alternatively wired bHLH transcription factor network to establish stomatal identity. Proc. Natl. Acad. Sci. USA, 113, 8326–8331. 27382177 10.1073/pnas.1606728113 PMC4961163

[tpj14560-bib-0084] Raissig, M.T. , Matos, J.L. , Gil, M.X. , ***et al*** **.** (2017) Mobile MUTE specifies subsidiary cells to build physiologically improved grass stomata. Science (New York, N.Y.), 355, 1215–1218. 10.1126/science.aal3254 28302860

[tpj14560-bib-0085] Raschke, K. (1976) How stomata resolve the dilemma of opposing priorities. Philos. Trans. Royal Soc. B Biol. Sci. 273, 551–560.

[tpj14560-bib-0086] Raschke, K. and Fellows, M.P. (1971) Stomatal movement in Zea mays: Shuttle of potassium and chloride between guard cells and subsidiary cells. Planta, 101, 296–316. 24488474 10.1007/BF00398116

[tpj14560-bib-0087] Raven, J.A. (2014) Speedy small stomata? J. Exp. Bot. 65, 1415–1424. 24609500 10.1093/jxb/eru032

[tpj14560-bib-0088] Richardson, F. , Brodribb, T.J. and Jordan, G.J. (2017) Amphistomatic leaf surfaces independently regulate gas exchange in response to variations in evaporative demand. Tree Physiol. 37, 869–878. 28898992 10.1093/treephys/tpx073

[tpj14560-bib-0089] Rockwell, F.E. , Holbrook, N.M. and Stroock, A.D. (2014) The competition between liquid and vapor transport in transpiring Leaves. Plant Physiol. 164, 1741–1758. 24572172 10.1104/pp.114.236323 PMC3982738

[tpj14560-bib-0090] Roth‐Nebelsick, A. (2007) Computer‐based studies of diffusion through stomata of different architecture. Ann. Bot. 100, 23–32. 17483152 10.1093/aob/mcm075 PMC2735294

[tpj14560-bib-0091] Rudall, P.J. and Knowles, E.V. (2013) Ultrastructure of stomatal development in early‐divergent angiosperms reveals contrasting patterning and pre‐patterning. Ann. Bot. 112, 1031–1043. 23969762 10.1093/aob/mct169 PMC3783234

[tpj14560-bib-0092] Schlüter, U. , Muschak, M. , Berger, D. and Altmann, T. (2003) Photosynthetic performance of an Arabidopsis mutant with elevated stomatal density (sdd1‐1) under different light regimes. J. Exp. Bot. 54, 867–874. 12554730 10.1093/jxb/erg087

[tpj14560-bib-0093] Schoch, P.‐G. , Zinsou, C. and Sibi, M. (1980) Dependence of the stomatal index on environmental factors during stomatal differentiation in leaves of Vigna sinensis L.: 1. Effect of light intensity. J. Exp. Bot. 31, 1211–1216.

[tpj14560-bib-0094] Schuler, M.L. , Sedelnikova, O.V. , Walker, B.J. , Westhoff, P. and Langdale, J.A. (2018) SHORTROOT‐mediated increase in stomatal density has no impact on photosynthetic efficiency. Plant Physiol. 176, 757–772. 29127261 10.1104/pp.17.01005 PMC5761779

[tpj14560-bib-0095] Shimazaki, K. , Doi, M. , Assmann, S.M. and Kinoshita, T. (2007) Light regulation of stomatal movement. Annu. Rev. Plant Biol. 58, 219–247. 17209798 10.1146/annurev.arplant.57.032905.105434

[tpj14560-bib-0096] Simkin, A.J. , McAusland, L. , Headland, L.R. , Lawson, T. and Raines, C.A. (2015) Multigene manipulation of photosynthetic carbon assimilation increases CO_2_ fixation and biomass yield in tobacco. J. Exp. Bot. 66, 4075–4090. 25956882 10.1093/jxb/erv204 PMC4473996

[tpj14560-bib-0097] Srivastava, A. , Lu, Z. and Zeiger, E. (1995) Modification of guard cell properties in advanced lines of Pima cotton bred for higher yields and heat resistance. Plant Sci. 108, 125–131.

[tpj14560-bib-0098] Tanaka, Y. , Sugano, S.S. , Shimada, T. and Hara‐Nishimura, I. (2013) Enhancement of leaf photosynthetic capacity through increased stomatal density in Arabidopsis. New Phytol. 198, 757–764. 23432385 10.1111/nph.12186

[tpj14560-bib-0099] Taylor, S.H. and Long, S.P. (2017) Slow induction of photosynthesis on shade to sun transitions in wheat may cost at least 21% of productivity. Philosophical Transactions of the Royal Society B: Biological Sciences, 372, 20160543. 10.1098/rstb.2016.0543 PMC5566890 28808109

[tpj14560-bib-0100] Vialet‐Chabrand, S.R.M. , Matthews, J.S.A. , McAusland, L. , Blatt, M.R. , Griffiths, H. and Lawson, T. (2017a) Temporal dynamics of stomatal behavior: modeling and implications for photosynthesis and water use. Plant Physiol. 174, 603–613. 28363993 10.1104/pp.17.00125 PMC5462030

[tpj14560-bib-0101] Vialet‐Chabrand, S. , Matthews, J.S.A. , Simkin, A.J. , Raines, C.A. and Lawson, T. (2017b) Importance of fluctuations in light on plant photosynthetic acclimation. Plant Physiol. 173, 2163–2179. 28184008 10.1104/pp.16.01767 PMC5373038

[tpj14560-bib-0102] Wang, C. , Liu, S. , Dong, Y. , Zhao, Y. , Geng, A. , Xia, X. and Yin, W. (2016) PdEPF1 regulates water‐use efficiency and drought tolerance by modulating stomatal density in poplar. Plant Biotechnol. J. 14, 849–860. 26228739 10.1111/pbi.12434 PMC11388919

[tpj14560-bib-0103] Woodward, F.I. (1987) Stomatal numbers are sensitive to increases in CO_2_ from pre‐industrial levels. Nature, 327, 617–618.

[tpj14560-bib-0104] Woolfenden, H.C. , Baillie, A.L. , Gray, J.E. , Hobbs, J.K. , Morris, R.J. and Fleming, A.J. (2018) Models and mechanisms of stomatal mechanics. Trends Plant Sci. 23, 822–832. 30149855 10.1016/j.tplants.2018.06.003

[tpj14560-bib-0105] Zoulias, N. , Harrison, E.L. , Casson, S.A. and Gray, J.E. (2018) Molecular control of stomatal development. Biochem. J. 475, 441–454. 29386377 10.1042/BCJ20170413 PMC5791161

